# National results and 6 years trends of nosocomial infection surveillance in French intensive care units (REA-RAISIN network)

**DOI:** 10.1186/2047-2994-4-S1-P236

**Published:** 2015-06-16

**Authors:** A Savey, A Machut, A Lepape, F L’heriteau, M Aupee, C Bervas, S Boussat, I Russell, K Chami, S Vaux, REA-Raisin Network

**Affiliations:** 1 UMR 5558, CNRS - UCBL1, France; 2 CClin Sud-Est, France; 3 GH Lyon Sud / HCL, Lyon, France; 4 CClin Paris-Nord, Paris, France; 5 CClin Ouest, Rennes, France; 6 CClin Sud-Ouest, Bordeaux, France; 7 CClin Est, Nancy, France; 8 Institut de Veille Sanitaire, Saint Maurice, France

## Introduction

Healthcare associated infections surveillance is a priority in intensive care units (ICUs). Since 2004, French national surveillance in adult ICUs (REA-RAISIN network) has targeted device-associated infections, for which control measures are essential.

## Objectives

To present 2014 national results and highlight the impact of prevention programmes over the last six years.

## Methods

Six months a year, ICUs collected data for each patient hospitalised more than 2 days. Surveillance focused on ventilation associated pneumonia (VAP), central venous catheter related infection or bacteraemia (CRI/CRB) and bloodstream infection (BSI) according to European protocol (ECDC). Analysis included patient’s characteristics, device exposures, ICU-acquired infections (microorganisms, antimicrobial resistance) and incidence ratios including ICU distributions and temporal trends (2009-2014). Multivariate analyses introducing the year of participation as a risk factor were performed for VAP and CRB.

## Results

In 2014, 212 ICUs included 34,226 patients and 10.7% presented at least one infection. Overall incidence rates were calculated: **14.26 VAP**/1,000 intubation-days, **3.53 BSI**/1,000 ICU-days, **0.66 CRI** and **0.51 CRB**/1,000 catheter-days.

In comparison with 2009, patients were more predisposed to infections due to significant evolution of their characteristics (age, SAPSII, antibiotic treatment at admission, immunosuppression) meanwhile device exposure decreased. All incidence rates of device-associated infections decreased significantly: VAP (-6.2%), CRI (-40.5%) and CRB (-43.3%), meanwhile decrease is not significant for BSI (-1.1%). Multivariate analysis confirmed this reduction in 2014 for VAP (adjusted OR: 0.90; CI_95_: 0.84-0.97) and CRB (adjusted OR: 0.56; CI_95_: 0.44-0.71).

## Conclusion

This surveillance network, including 50.4% of French ICU beds, represents a national reference and appears effective in describing and monitoring infectious risk in ICUs. The **significant decrease in 2014 for VAP and CRB** can be related to practice improvement and higher level of patient safety.

## Disclosure of interest

None declared.

